# A new species of the genus *Ambrostoma* Motschulsky (Coleoptera, Chrysomelidae, Chrysomelinae) from South Korea, with larval descriptions and biological notes

**DOI:** 10.3897/zookeys.321.4972

**Published:** 2013-08-02

**Authors:** Hee-Wook Cho, Lech Borowiec

**Affiliations:** 1 Department of Biodiversity and Evolutionary Taxonomy, University of Wrocław, Przybyszewskiego 63/77, 51-148 Wrocław, Poland

**Keywords:** Chrysomelidae, *Ambrostoma*, new species, larva, biology, ovoviviparity, Korea

## Abstract

*Ambrostoma koreana* **sp. n.** is described from South Korea. Detailed descriptions and illustrations of adult and all larval instars are provided and differences to closely related species are discussed. Ovoviviparity is reported for the first time in the genus *Ambrostoma*. Notes on distribution, host plant and biology are also provided.

## Introduction

The genus *Ambrostoma* was described by Motschulsky (1860) for three species from Russia (Amur region), China and Nepal, respectively, with *Ambrostoma quadriimpressum* (Motschulsky, 1845) [= *Ambrostoma superbum* (Thunberg, 1787)] as its type species. Since then, nine species have been described in East Asia and the Himalayas (Achard 1922, Chen 1934, 1936, Gressitt and Kimoto 1963, Wang 1992, Kimoto and Osawa 1995, Medvedev 2007). Chen (1936) divided the genus into two subgenera, *Ambrostoma* and *Parambrostoma*, based on elytral punctures, setae on elytral epipleura and length ratio of antennomeres, and later Wang and Chen (1981) raised these subgenera to the generic status. These two genera were recently revised to include fifteen species and the monophyly of both genera was well supported by morphological characters of adults (Ge et al. 2012). *Ambrostoma* is widely distributed in East Asia and comprises eight species. Members of the genus are characterized by a distinct transverse depression near the base of elytra, inner margin of elytral epipleura with setae along an entire length, procoxal cavities open posteriorly, metasternal process immarginate apically and tarsal claws simple. The larva are only known and for only one species of the genus: *Ambrostoma superbum* which is a major pest in China, has been described by Medvedev and Voronova (1976). *Ambrostoma* larva is easily recognized among the subfamily Chrysomelinae by their orange stripes in live specimens, undeveloped tubercles, a dark pattern on the dorsum and black markings on the head and pronotum.

In 2006, the first author collected unusual *Ambrostoma* specimens, adults and larvae, on Namhaedo Island in South Korea. After a detailed examination we concluded that they belong to a new species described below. We compared the larval morphology of *Ambrostoma* to those of *Parambrostoma*. Notes on distribution, host plant, biology and occurrence of ovoviviparity are also provided.

## Material and methods

The type specimens were deposited in the Department of Biodiversity and Evolutionary Taxonomy, University of Wrocław, PolandDBET and H.-W. Cho’s private collection, Republic of KoreaHWC. Biological observations were made in April–May 2006, at the type locality and under laboratory conditions. Adults and larvae collected from the type locality were kept in plastic containers (10 cm diameter, 12 cm deep) with leaves of host plant and checked daily. All larval specimens used in the study were preserved in 70% ethanol. For morphological studies of minute structures, some larvae were dissected, cleared in 10% sodium hydroxide solution, rinsed in distilled water, and then mounted on slides with Swan’s liquid (20 g distilled water, 15 g gum arabic, 60 g chlorhydrate, 3 g glucose, and 2 g glacial acetic acid). Genitalia were dissected from adult specimens softened in plastic containers with wet tissue paper for 12–24 hours. The aedeagus was softened in 10% sodium hydroxide solution for 6–12 hours and placed in distilled water. The careful insertion of a sharp-pointed thick nose hair and injection of 5% ethanol into the foramen of aedeagus were repeated until the internal sac was fully everted. After washing with absolute ethanol, the genitalia were preserved in a microvial with glycerin and pinned with the specimen. Descriptions and illustrations were prepared using Nikon SMZ800 and Nikon Eclipse E600 microscopes, each equipped with a drawing tube. Habitus images were taken by a Nikon Coolpix 4500 digital camera attached to a Nikon SMZ1500 microscope. The letters L, S and M in parentheses signify long, short, and micro setae, respectively.

## Results

### Ambrostoma koreana

Cho & Borowiec sp. n.

http://zoobank.org/4FEAAE6E-87B0-4AFA-B40F-3DAADB68CF75

http://species-id.net/wiki/Ambrostoma_koreana

Figs 1 –20

#### Type locality.

South Korea: Gyeongnam Province, Namhaedo Island, Mangunsan Mountain, 34°51'52"N, 127°51'47"E.

#### Type material.

Holotype: male (DBET), KOREA: Gyeongnam Province, Namhaedo Island, Mangunsan Mountain, 34°51'52"N, 127°51'47"E 280 m, 7.IV.2006, H.-W. Cho. Paratypes: 2 males and 6 females (DBET), same data as holotype; 10 males and 10 females (HWC), same data as holotype except for 13.VI.2007, H.-Y. Kwon; 1 male (HWC), KOREA: Gyeongnam Province, Miryang, Gajisan Mountain, 35°35'46"N, 128°59'38"E 500 m, 17.VIII.2001, T.-H. Ahn; 1 female (HWC), same data as preceding paratype except for 25.V.2001, G.-S. Jung.

#### Other material.

42 larvae collected or obtained from adults, same data as holotype except for 23.IV.–15.V.2006.

#### Adult

(Figs 1–7, 19).

**Figures 1–2.**
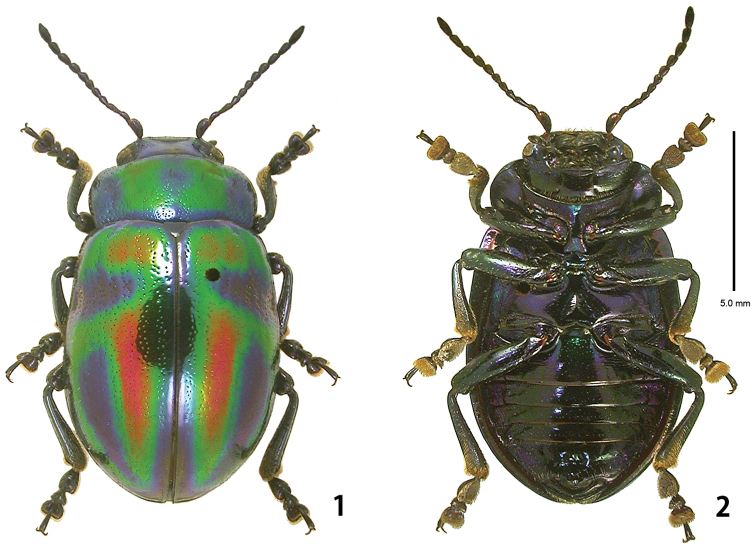
Habitus of *Ambrostoma koreana* sp. n., holotype. **1** dorsal view **2** ventral view.

**Figures 3–7.**
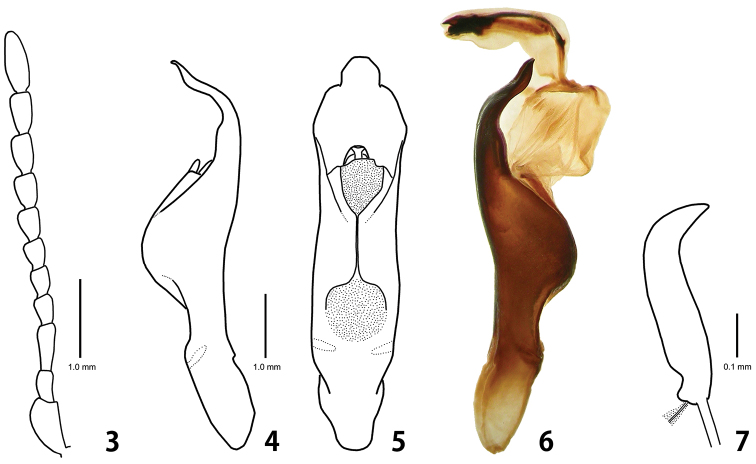
Adult of *Ambrostoma koreana* sp. n. **3** antenna **4** aedeagus lateral view **5** aedeagus dorsal view **6** aedeagus with everted internal sac laternal view**7** spermatheca.

**Diagnosis.** The new species is very similar in coloration to *Ambrostoma leigongshana* Wang, but can be distinguished by the following characters: antennomere 3 much longer than 4 (equal in length in *Ambrostoma leigongshana*); pronotum with moderately dense punctures in lateral depression (dense and coarse punctures in *Ambrostoma leigongshana*); aedeagus widest at apical 1/5, thence narrowed with trapezoidal apex (elongate apically with rounded apex in *Ambrostoma leigongshana*); spermatheca almost straight, curved at apex (strongly curved in *Ambrostoma leigongshana*).

**Description. Holotype** (Figs 1–2). Body length 11.7 mm, width 5.7 mm, strongly convex dorsally. Head emerald green to ultramarine with 2 pairs of small orange yellow markings on central part. Mouthparts midnight blue. Antennomeres 1–7 ultramarine, 8–11 black. Pronotum emerald green to ultramarine with a pair of large orange yellow markings. Scutellum ultramarine. Elytra ultramarine with a pair of orange yellow markings at base and 2 pairs of orange yellow longitudinal markings on central-posterior part, all markings surrounded by emerald green. Venter mainly ultramarine with greenish luster. Legs midnight blue.

*Head*. Width 3.4 mm, interocular distance 2.5 mm. Vertex and frons with sparse and small punctures. Clypeus and labrum with small punctures bearing long setae. Mandibles with moderately dense punctures bearing setae on outer surface. Maxillary palp 4-segmented with apical palpomere distinctly widened and truncate. Antennae (Fig. 3) reaching elytral humeri; antennomere 1 robust, longer than 3; antennomere 2 as long as 4; antennomere 3 distinctly longer than 4; antennomeres 5–10 moderately widened; antennomere 11 longest and 2.6 times as long as wide.

*Pronotum*. Length 2.7 mm, width 5.4 mm. Lateral sides roundly widened anteriorly, widest at anterior 1/4. Anterior margin widely emarginated. Trichobothria on anterior and posterior angles. Disc with dense and small punctures, larger than those of head; hardly confluent large punctures in lateral longitudinal depression; interspaces with spare and minute punctures. Scutellum subtriangular, slightly wider than long, impunctate.

*Elytra*. Length 8.8 mm, width 6.8 mm. Sides moderately widened posteriorly and widest at posterior 2/5, thence rounded at apex. Disc with double irregular rows of punctures; transverse depression with large punctures, subequal to those of side of pronotum; interspaces with spare and minute punctures. Epipleuron flat, inner margin with micro setae along an entire length. Hind wing well developed.

*Venter*. Hypomera impunctate; prosternum with sparse and small punctures; prosternal process strongly enlarged and slightly emarginated apically. Abdominal sternites wrinkled laterally with sparse and small punctures; last abdominal sternite deeply emarginated on both sides. Legs moderately robust; tibiae simple without preapical tooth; fore and middle legs with tarsomere 1 slightly narrower than 3; tarsal claws simple.

*Aedeagus*. Strongly convex at middle, curved and sinuated at apex in lateral view (Fig. 4); subparallel-sided, widest at apical 1/5, thence narrowed with trapezoidal apex, with 2 weakly sclerotized plates in dorsal view (Fig. 5); internal sac curved, shorter than median lobe with thick and long flagellum (Fig. 6).

**Paratypes.** Body length 10.9–12.7 mm, width 5.2–6.2 mm. Coloration similar to holotype. Female: larger than male; tarsomere 1 of fore and middle legs distinctly narrower than 3; last abdominal sternite rounded; spermatheca (Fig. 7) almost straight, curved at apex.

#### First instar larva

(Figs 15, 17).

**Description.** Body length 2.7–4.2 mm, width 1.2–1.7 mm, head width 0.90–0.95 mm (n = 5). Body convex dorsally on abdomen. Pale yellow with head dark brown, tubercles and legs brown (Fig. 17). Tubercles weakly developed (Fig. 15). Sclerotized platelets on dorsum dense and strong, on venter almost absent. Setae longer than in other instars, bases of setae sclerotized.

*Head*. Vertex and temporal side with 30–32 pairs of long setae and 15–19 pairs of short setae. Frons with 16–19 pairs of long setae. Clypeus and labrum, each with 2 pairs of long setae. Mouthparts similar in shape and chaetotaxy to those of the last instar larva, except for stipes with 3 setae and postmentum with 3 pairs of setae.

*Thorax*. Prothorax with D-DL-EP (53–57L 4–6S); P (1L); ES (1L) weakly sclerotized; SS represented by a short seta; sternal region with 1–2 additional setae. Meso- and metathorax with Dae (1L); Dpi (1L); dorsal region with 11–13 additional short setae; DLi (1–3L) well developed with egg burster; DLe (4–5L 8–11S); EPa (4–5L 5S) fused with spiracle; EPp (2L 3–4S); P (1L); ES (1L) weakly sclerotized; SS represented by a short seta; sternal region with 2–3 additional setae.

*Abdomen*. Dorsal and dorsolateral regions with 31–35 short setae; Dpi (1L); Dpe (1L); DLp (1L); EP (9–10L 8–10S); P (3S) divided; PS-SS represented by 4–5 short setae; ES (1S); segment 8 with D-DL (3–4L 11–12S) fused; segment 9 with D-DL-EP (3L 11–12S) fused; segment 10 with pygopod developed; egg burster on segment 1 much smaller than thoracic ones.

#### Second instar larva.

#### Description.

Similar to the last instar larva except for following characters: body length 4.2–5.7 mm, width 1.8–2.4 mm, head width 1.25–1.35 mm (n = 4); black markings on head and dark patterns on dorsum much larger than those of the last instar larva; pronotum with black markings partly connected.

#### Third instar larva.

**Description.** Similar to the last instar larva except for following characters: body length 7.2–8.1 mm, width 2.8–3.8 mm, head width 1.90–2.05 mm (n = 3); dark patterns on dorsum much larger than those of the last instar larva.

#### Fourth (last) instar larva

(Figs 8–14, 16, 18).

**Figures 8–14.**
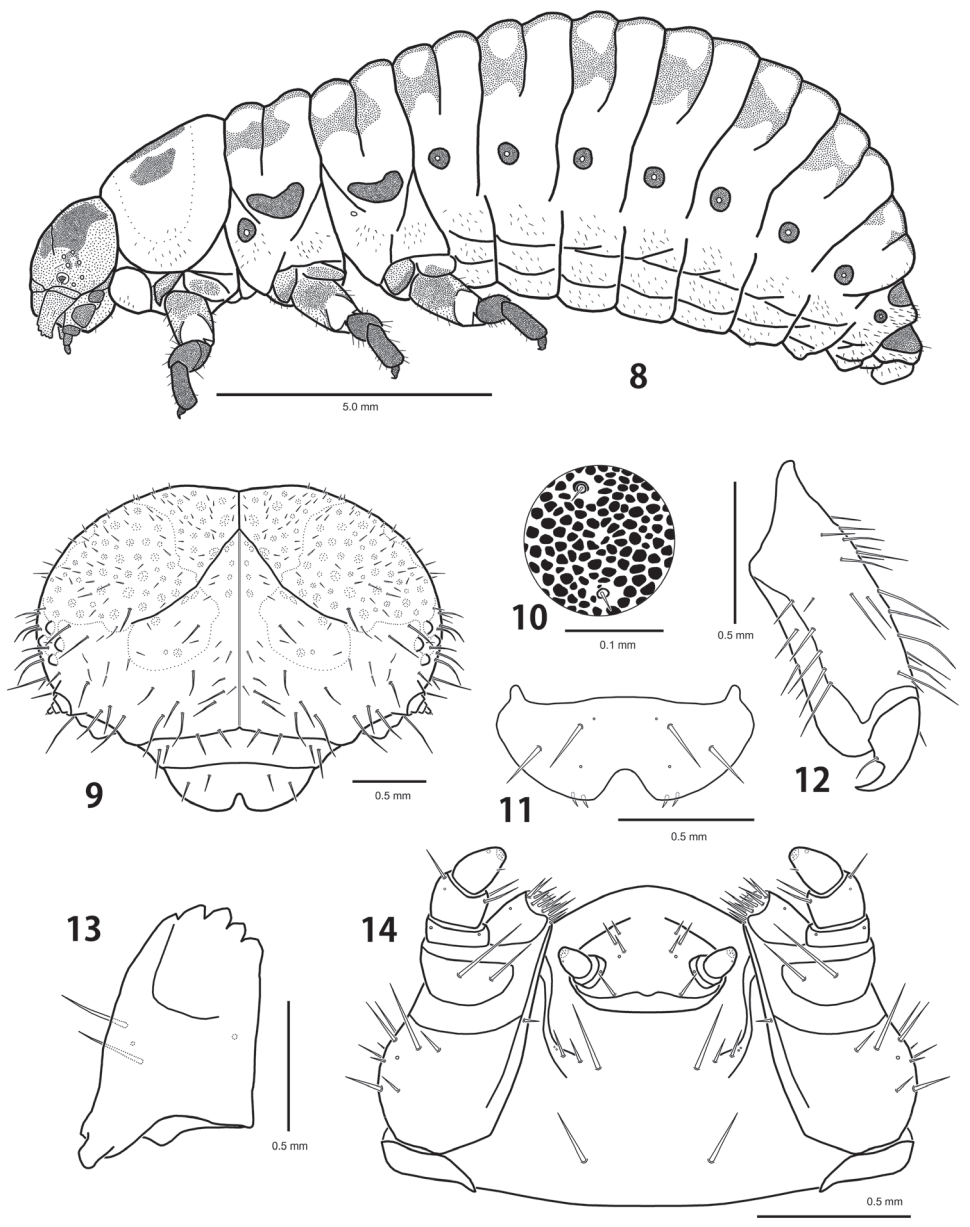
Last instar larva of *Ambrostoma koreana* sp. n. **8** habitus **9** head **10** sclerotized platelets on dorsal mesothorax **11** labrum **12** tibia and tarsungulus **13** mandible **14** lower mouthparts.

**Figures 15–16.**
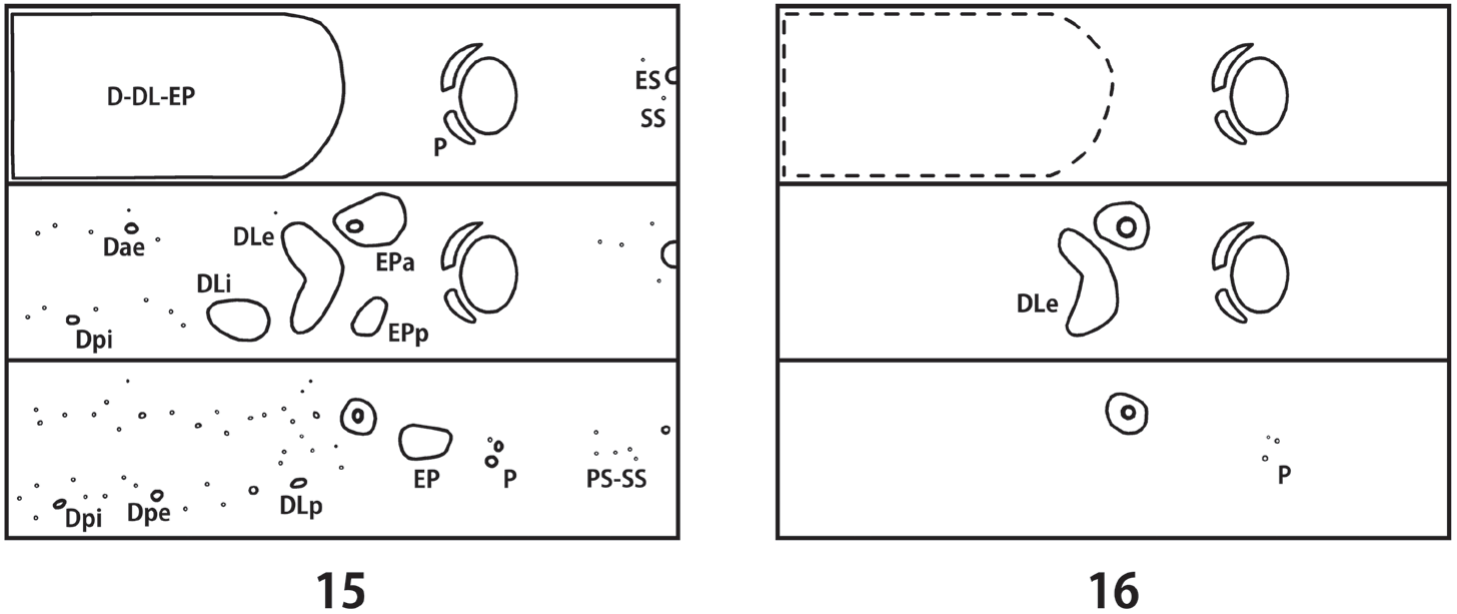
Schematic presentation of tubercular patterns (top: prothorax, middle: mesothorax, bottom: 2nd abdominal segment). **15** first instar larva **16** last instar larva.

**Figures 17–18.**
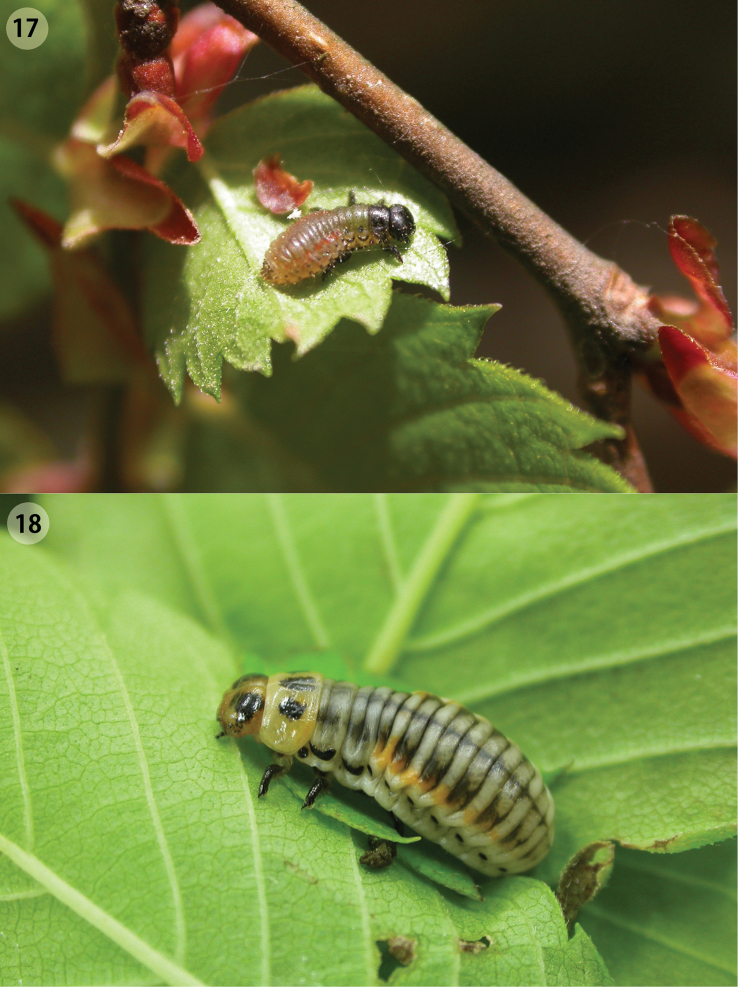
Larvae of *Ambrostoma koreana* sp. n. on leaves of *Zelkova serrata*. **17** first instar larva **18** last instar larva.

**Figure 19.**
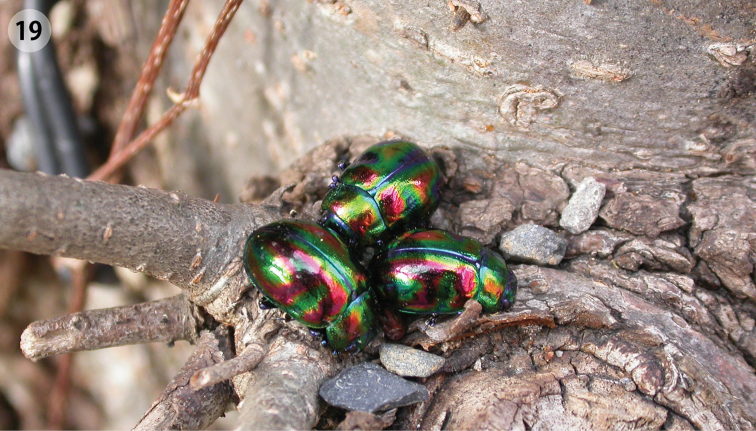
Overwintered adults of *Ambrostoma koreana* sp. n.

#### Diagnosis.

The last instar larva is easily distinguished from larva of *Ambrostoma superbum* (Thunberg) in the following characters: pronotum with 3 black markings (2 black markings in *Ambrostoma superbum*); dorsolateral posterior region of each abdominal segment without dark patterns (with dark patterns in *Ambrostoma superbum*); width of peritreme less than half width of abdominal segment (equal to half width in *Ambrostoma superbum*).

#### Description.

Body length 11.6–12.8 mm, width 4.7–5.3 mm, head width 2.60–2.65 mm (n = 5). Body strongly convex dorsally on abdomen (Fig. 8). Yellowish white in alcohol specimens, creamy white with orange stripes in live specimens (Fig. 18). Head yellowish brown with a pair of large black markings. Pronotum with 3 black markings. Tubercles, spiracles and legs dark brown. Dorsum with dark patterns consisting of dense and strong sclerotized platelets, sparsely covered with micro setae (Fig. 10). Tubercles undeveloped (Fig. 16). Venter covered with short and moderately long setae, bases of setae not sclerotized.

*Head*. Hypognathous, rounded, well sclerotized, covered with dark spots (Fig. 9). Vertex and temporal side with 10–11 pairs of long setae and 77–82 pairs of short setae. Epicranial suture Y-shaped; coronal suture distinct along an entire length; frontal suture indistinct for lateral 2/5. Frons slightly depressed medially with 14–15 pairs of long setae and 17–22 pairs of short setae. Endocarina distinct; epistomal suture developed. Six stemmata on each side. Antenna 3-segmented; antennomere 1 without seta; antennomere 2 with a sensory papilla and 4 setae; antennomere 3 with 5 setae. Clypeus trapezoid with 2 pairs of setae. Labrum (Fig. 11) deeply emarginate anteriorly with 2 pairs of setae; epipharynx with 2 pairs of setae. Mandibles (Fig. 13) symmetrical, 5-toothed with 2 setae. Maxillary palp (Fig. 14) 3-segmented; palpomere 1 without seta; palpomere 2 with 3 setae; palpomere 3 conical with a seta; palpiger with 2 setae; mala with 13–14 setae; stipes with 10–13 setae; cardo without seta. Labial palp 2-segmented; prementum with 4–5 pairs of setae; postmentum with 4 pairs of setae.

*Thorax*. Prothorax with D-DL-EP scattered with micro setae, not sclerotized; P (6–7S); ES and SS, each represented by a short seta; sternal region with 14–16 additional setae. Meso- and metathorax with DLe (27–30M); P (8–10S); EPa and EPp, each represented by 23–27 short setae; ES and SS, each represented by a long seta; sternal region with 20–35 additional setae; mesothoracic spiracle annuliform with large peritreme; metathoracic spiracle vestigial. Legs rather stout; tibia with 28–30 setae; tarsungulus strongly curved; basal tooth weakly developed with a seta (Fig. 12).

*Abdomen*. Tubercles absent on segments 1–7 except for P represented by 3–4 short setae arising from sclerotized bases; epipleural to sternal regions with a lot of short setae. Segment 8 with D-DL (10–12S); segment 9 with D-DL-EP (12–14L 1–2M); segment 10 with pygopod well developed; spiracle with large peritreme similar to mesothoracic one, but smaller; eversible glands absent.

**Figure 20.**
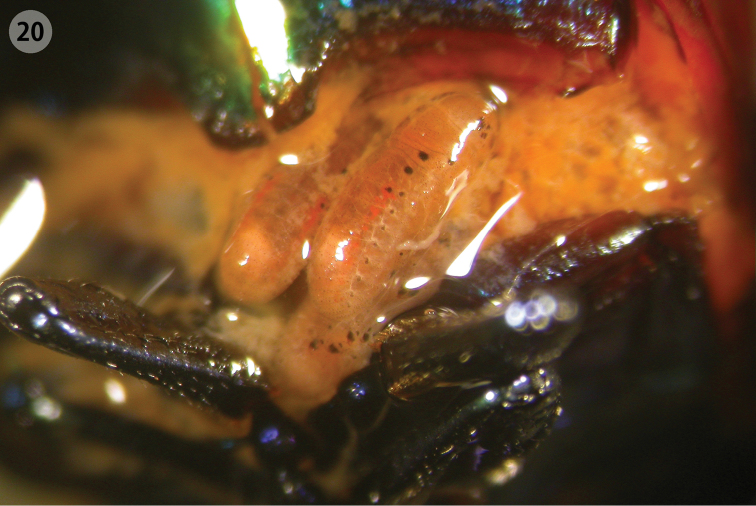
Larvae inside the abdomen of female of *Ambrostoma koreana* sp. n.

#### Etymology.

This endemic species is named after the type locality, Korea.

#### Distribution.

Specimens were collected in the southeastern part of the Korean Peninsula and on adjacent Namhaedo Island.

#### Biological notes.

Overwintered adults were observed under bark or in fallen leaves near the base of trees in early April (Fig. 19). Adults and larvae fed on leaves of *Zelkova serrata* Makino (Ulmaceae) which is quite a common tree in Korea. *Ulmus pumila* Linnaeus (also Ulmaceae) was the only known host plant for three other *Ambrostoma* species (Yu et al. 1996). On 7 May 2006, a female laid 4 eggs that contained fully developed embryos in the laboratory. Four females were dissected shortly afterward, and 17–79 enclosed larvae were found in abdomen (Fig. 20). About 50 ovoviviparious species are actually known in 7 genera of Chrysomelinae (Bontems 1988, Bontems and Lee 2008). Ovoviviparity is reported for the first time in the genus *Ambrostoma*.

### Key to the adults of *Ambrostoma* species (modified from Ge et al. 2012)

**Table:** 

1	Punctures of transverse impression of elytra subequal to those of the lateral sides of pronotum	2
–	Punctures of transverse impression of elytra much finer than those of sides of pronotum	3
2	Elytra with 3 broad transverse bands of purplish copper-red	*Ambrostoma rugosopunctatum* Chen
–	Elytra with 1 transverse and 4 longitudinal bands of orange yellow	*Ambrostoma koreana* sp. n.
3	Elytra with large well-defined post-median violaceous patch surrounded by green	4
–	Elytra without well-defined post-median violaceous patch surrounded by green	5
4	Arrangement of elytral punctures almost entirely irregular; last abdominal sternite of male deeply emarginate on both sides	*Ambrostoma fulgurans* (Achard)
–	Elytral punctures arranged in simple rows at base before transverse impression, slightly irregular beyond impression; last abdominal sternite moderately sinuate on both sides	*Ambrostoma fasciatum* Chen
5	Pronotum slightly dilated in front of middle region; interspaces between elytral striae finely and densely punctuate	*Ambrostoma superbum* (Thunberg)
–	Pronotum strongly dilated in front of middle region; interspaces between elytral striae finely and very sparsely punctuate	6
6	Elytra with single striae	*Ambrostoma chinkinyui* Kimoto & Osawa
–	Elytra with double striae	7
7	Pronotum with dense and coarse punctures in lateral depression	8
–	Pronotum with sparse punctures in lateral depression	*Ambrostoma omeishanum* Gressitt & Kimoto
8	Elytra with longitudinal purple markings on media posterior surrounded by green; last abdominal segment in male shallowly sinuate	*Ambrostoma leigongshanum* Wang
–	Elytra with 4 purple longitudinal stripes on media posterior part; last abdominal segment in male deeply sinuate	*Ambrostoma fortunei* (Baly)

## Discussion

The *Ambrostoma* species has not been recorded in Korea since Chen (1936) mentioned *Ambrostoma superbum* without any locality data and comments. *Ambrostoma superbum* is widely distributed in Southeastern Siberia, Eastern Mongolia, Northeastern China and probably North Korea (Kippenberg 2010, under name *Ambrostoma quadriimpressum quadriimpressum*), whereas *Ambrostoma koreana* is restricted to Namhaedo Island and Miryang, Gyeongnam Province, South Korea. The first instar larva of *Ambrostoma* was described for the first time. The second and third instar larvae are very similar in shape and coloration to the last instar larva, but the first instar larva distinctly differs in having more developed tubercles and long setae. The tubercular pattern of the first instar larva was similar to some larvae of the genus *Chrysolina* Motschulsky. Based on body shape, tubercular pattern and biology, *Ambrostoma* larva belongs to the generic group *Chrysolina* proposed by Kimoto (1962). The larva of *Parambrostma mahesa* (Hope) also belongs to the generic group *Chrysolina* (Takizawa 1989). The monophyly of both genera, *Ambrostoma* and *Parambrostoma* carried out by Ge et al. (2012), was supported by larval characters. The mature larvae of these two genera are similar in having undeveloped dorsal tubercles, sclerotized platelets and sparse micro setae on dorsum. However, the considerable differences can be summarized as follows: dark patterns present on dorsum in *Ambrostoma* and absent in *Parambrostoma*; head and pronotum are pale coloured with black markings in *Ambrostoma* and entirely black in *Parambrostoma*; prothoracic and ventral tubercles are not scleotized in *Ambrostoma*, whereas they are well scleotized in *Parambrostoma*; basal tooth of tarsungulus in *Ambrostoma* is weakly developed while in *Parambrostoma* it is strongly developed.

## Supplementary Material

XML Treatment for Ambrostoma koreana
